# Chocolate scents and product sales: a randomized controlled trial in a Canadian bookstore and café

**DOI:** 10.1186/s40064-016-2303-5

**Published:** 2016-05-21

**Authors:** Mary C. McGrath, Peter M. Aronow, Vivien Shotwell

**Affiliations:** Department of Political Science, Yale University, New Haven, CT USA; Department of Biostatistics, Yale School of Public Health, New Haven, CT USA; Trident Bookstore and Café, Halifax, NS Canada

**Keywords:** Atmospherics, Ambient scent, Purchasing behavior, Book sales

## Abstract

We report the results of a 31-day trial on the effects of chocolate scent on purchasing behavior in a bookstore. Our study replicates and extends a 10-day randomized controlled trial in order to examine the generalizability of the original finding. We first introduce the study of store atmospherics and highlight the importance and dearth of replication in this area. In the next section, we describe the original study and discuss the theory of ambient scent effects on product sales, and the role of scent-product congruity. We then describe our design and methods, followed by presentation and discussion of our results. We find no evidence that chocolate scent affects sales. These findings indicate the importance of replication in varied settings. Contextual factors and the choices available to customers may moderate the effects of ambient scent on purchasing behavior. Our study highlights the value of examining the generalizability of experimental findings, both for theory and practice.

## Background

Careful composition of a store’s atmosphere, according to a stream of literature in consumer research, can tip the balance between success and failure for a business (Turley and Milliman 2000). Experimental investigation of optimal store atmospherics has a long history: 50 years ago Smith and Curnow ran a field experiment manipulating the volume of music in a supermarket (Smith and Curnow 1966). A large menu of controllable atmospheric variables (e.g., music, lighting, wall color, ambient scent) each of which can take on innumerable values (e.g., chocolate scent, citrus scent, sea breeze scent), and a continuously regenerating array of product-types on which to test the effects has allowed the development of a large experimental literature in store atmospherics. With such a wealth of potential stimuli—Turley and Milliman list 57 different categories of atmospheric variable—there is always a new atmospheric effect to investigate, and replication studies are rare.

Turley and Milliman’s excellent review of the experimental literature makes no reference to replication studies. In a more recent, critical review focused on the effects of ambient scent, Teller and Dennis (2012) conduct a replication of Chebat and Michon (2003)—to our knowledge the only published replication study on ambient scent atmospherics.^1^ The study of store atmospherics represents a realm of research with clear substantive implications and potentially meaningful economic effects. The generalizability and replicability of experiments like these matter both for scientific reasons and for economic reasons.

In recent years, a crisis of replicability in many scientific disciplines has drawn greater attention to the importance of replication in establishing a reliable understanding of a stimulus and behavioral response (Schooler 2014). And replication and extension take on an additional layer of import when findings carry actionable implications for practitioners, as in a field like consumer research. Because experimentation is widely recognized as the gold-standard, publicized experimental findings in consumer research are likely to be taken as recommended practice. In hopes of achieving the results suggested by experimental findings, business owners may invest scarce resources to adopt practices that may prove unreliable or unsuitable upon repeated testing.

This study reports results from an ambient scent field experiment that replicates and extends the experiment conducted by Doucé et al. (2013). Doucé et al. conducted a randomized 10-day trial in a Belgian chain bookstore examining the effects of ambient chocolate scent on consumer behavior. Doucé et al. note that total sales increased 5.07 % on days in which the chocolate scent was administered, and the reported increase in sales was publicized widely in the popular press (see, e.g., Dooley 2013) and promoted in trade publications (see, e.g., Abrams 2013). The experiment reported here aimed to assess the generalizability of the Doucé et al. results by extending the design to a common variant of the original setting: a bookstore with adjoining café area. We test whether the finding of increased sales holds when products in the same domain as the chocolate scent—coffee and food items—are offered for sale alongside books. Our experiment found no effect of chocolate scent on either bookstore or café sales.

### Ambient scent and bookstore purchasing behavior

The primary findings of Doucé et al. pertain to customers’ “approach behavior”, or engagement with products (e.g., “closely examining multiple books”). The authors categorized two genres of books as congruent with chocolate smell (*Food & Drink [Cook] Books* and *Romance Novels & Romantic Literature*), and two genres as incongruent (*History* and *Crime, Thrillers & Mystery*). Doucé et al. recorded an increase in both incongruent book sales (22 % increase) and congruent book sales (40 % increase) on days in which the chocolate scent was administered. The authors do not report measures of the uncertainty around these estimates. In their main finding, the authors report an increased likelihood of purchasing a congruent-category book over an incongruent-category book. Doucé et al. caution that countervailing effects on congruent and incongruent product types may pose a challenge for stores that sell a variety of products: “Retailers offering more than one product type should be aware of the possible negative effects of a pleasant scent that is thematically incongruent with part of the store offerings” (Doucé et al. 2013, p. 69).

Schifferstein and Blok (2002) describe the potential mechanisms through which the thematic associations of an odor may influence consumer behavior. If a stimulus scent is unconsciously detected, odor priming will increase the accessibility of knowledge related to the stimulus. If the scent is consciously detected but unidentified, then the processes of trying to place the scent will generate associations—some correct and related to the stimulus, others incorrectly associated with the stimulus, as the actual identity of the scent remains unknown. Finally, a scent may be consciously detected and identified, in which case the scent activates only one’s true associations with the stimulus, which in turn may generate search behavior for related products.

The behavioral result of each of these three modes of influence depends on whether the stimulus scent is congruent with the products offered for sale (i.e., the thematic associations of the scent are related to the products) or incongruent (the thematic associations are unrelated to the products in question). Foreshadowing Doucé et al.’s concluding caveat, Schifferstein and Blok propose countervailing effects dependent on congruency: “…if some products in a store are thematically congruent with the ambient smell in that store whereas others are not, sales of the congruent products may benefit from the smell, whereas sales of the incongruent products may be hampered by it” (p. 541). Testing this hypothesis on magazine sales in three bookstores, Schifferstein and Blok found no evidence of countervailing effects: ambient scents neither increased sales of magazines deemed congruent with the scent nor decreased sales of magazines deemed incongruent.

Doucé et al. note that they selected a bookstore with no café area, and no nearby “shops associated with scents (e.g., a coffeehouse)” (p. 67). Bookstores frequently include an area serving coffee and pastries, making the bookstore with café arrangement a natural context in which to test the generalizability of Doucé et al.’s finding of an ambient scent effect on consumer behavior. We test whether a chocolate scent released on randomly assigned days had an effect on either product-type in the consumer’s choice set: books or café items. This experiment thus examines whether the contextual factors of the store and the particular choice set available to customers moderates the effect of a chocolate scent on purchasing behavior. Extending the study to a slightly different setting, we gain insight into the generalizability of an effect of chocolate scent on bookstore sales.

## Methods

We conducted a trial over 31 days in an 800-square-foot independent bookstore in Canada. The bookstore adjoined a café; in addition to books, the store sold greeting cards, espresso drinks, pastries, and loose coffee and tea. Apart from the owners of the bookstore (who administered the trial), the staff were not made aware of the trial, nor were the customers.

The trial used a between-subjects design in which the 31 experimental days were assigned one of two conditions: chocolate scent dispersion (treatment) and no scent (control). Condition was determined by Bernoulli (coin flip) random assignment with probability .5 independently across days. Chocolate scent was dispersed for a full day on randomly assigned treatment days (from 9:30 a.m. until 5:30 p.m. on weekdays, 9:30 a.m. until 5:00 p.m. on Saturdays, and 11 a.m. until 5:00 p.m. on Sundays), and was not dispersed on control days.

The aroma was dispersed by two methods, one at each end of the bookstore. Chocolate essential oil (Theobroma cacao 100 % Pure Extract) was obtained from Ananda Aromatherapy in Boulder, CO. Near the entrance of the bookstore, an electric scent diffuser designed for continuous scent release was used to warm and disperse the essential oil throughout operating hours, diffusing approximately .25–.30 ml of the liquid scent over the course of a treatment day. To intensify the treatment and ensure that a chocolate scent was present through the entire store, melted dark chocolate was maintained over a low heat source in an exposed metal pan at the other end of the bookstore. Both the scent diffuser and the pan-diffused chocolate were out of the customers’ line of vision. No other alterations to the store atmosphere, personnel, or layout were made during the course of the 31 experimental days, nor were any special promotions run during the experiment.

The primary goal of the present study was to test whether Doucé et al.’s finding extended to a setting in which a café area adjoined the bookstore. In addition to this variation in the environment which is the main focus of the study, two differences between the original study and this replication and extension should be noted. First, the setting in our study was an independent bookstore rather than a chain, with a total size of around one-third the area of the chain bookstore in the original study. Our replication study is based on three times as many observations (experimental days) as the original study, but the volume of traffic in our site is smaller: an average of 21 customers per day during the experimental period, as opposed to roughly 100 customers per day in the original study. Second, for cost considerations, the independent bookstore owner chose to use pan-diffused chocolate as a secondary scent source rather than purchasing a second electric scent diffuser.

Sales data were recorded in three categories: book sales, café sales (pastries, coffee, and tea), and bulk sales (primarily coffee beans, but also loose tea and spices). Approval for obtaining sales records was granted by the bookstore and authorized by the Yale University Institutional Review Board. Full replication data and code are available at the Yale Institution for Social and Policy Studies data archive (http://isps.yale.edu/research/data).

## Results and discussion

A manipulation check modeled after Doucé et al. was conducted to evaluate four aspects of the scent: spontaneous detection, prompted detection, spontaneous identification, and recognition of the scent. Fifteen customers were asked to respond to questions about the store atmosphere. To assess spontaneous detection, customers were asked whether they noticed something special in the store atmosphere, with responses coded as 1 if the customer mentioned an ambient scent, 0 otherwise. If the customer did not volunteer that a scent was present, ambient scent was mentioned and the subject was asked whether they could detect a scent now that scent was mentioned. Subjects were then asked if they could identify the scent. Subjects who did not spontaneously identify the scent as chocolate were asked if they recognized the scent as chocolate.

We maintain the intensity of the treatment scent at a stronger level than in the original study to avoid the possibility of the treatment scent being overwhelmed by scents from the café. As noted above, the theory behind the signal function of ambient scent holds that a consciously detected and identified scent should activate one’s true associations with the stimulus and thus promote search behavior for related products (Schifferstein and Blok 2002). In Doucé et al., the intensity of the scent was lowered until the researchers obtained a sub-sample of surveyed customers in which no respondents spontaneously detected a scent, but all recognized the scent as chocolate once it was mentioned as such (the size of this sub-sample is not reported in Doucé et al.). Our objective was to maintain a detectable ambient scent; we did not seek to eliminate spontaneous detection of the chocolate scent.

In our sample of 15 customers, six spontaneously detected a scent. Once prompted to consciously attend to scent, all but one of the respondents were able to discern the presence of an ambient scent. Six respondents spontaneously identified that ambient scent as chocolate, and another five reported recognizing the scent as chocolate once the enumerator identified the scent as such. The total proportion of our sample reporting a detectable ambient scent was .93 (95 % CI: .81, 1.06). One limitation of the small sample size in our manipulation check is that the confidence intervals for our estimates are relatively wide. However, since the lower bound of our estimate indicates that at least 80 % of patrons would discern the presence of an ambient scent, we remain confident that the scent was sufficiently detectable.

We present two regression-based analyses of the trial. The first is a simple ordinary least squares regression of our four outcomes: total sales, bulk sales, café sales, and book sales in Canadian dollars (CAD). This strategy is logically equivalent to taking a simple mean difference between treatment and control. To improve efficiency, the second strategy uses ordinary least squares adjusting for a mean-centered linear time trend as well as mean-centered fixed effects for the day of week, following Lin (2013). In all cases, robust standard errors are used to estimate standard errors, with *p* values computed under a normal approximation.

**Fig. 1:**
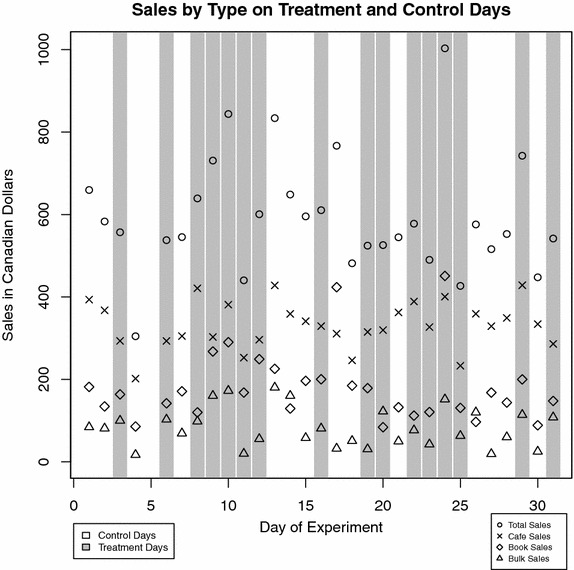
Sales by type on treatment and control days. All sales data associated with the randomized controlled trial. The x-axis shows each of the 31 days of the experiment, with *gray vertical bars* indicating days on which the chocolate scent was administered. The y-axis shows sales in Canadian dollars (CAD). The *markers* indicate sales per day: *circles* denote total sales, *Xs* denote café sales, *diamonds* denote book sales, and *triangles* denote bulk coffee bean/loose tea/spice sales

We present the complete raw results of the trial in Fig. 1. This figure shows the movement of sales over time and the relationship between sales and the randomized administration of the chocolate scent. Gray bars indicate the randomly assigned treatment days. The markers show sales per day, with sales type indicated by the marker symbols: circles show total sales, Xs show café sales, diamonds show book sales, and triangles show bulk sales. Visually, there is little evidence to suggest that the chocolate scent has any effect on outcomes.

In Table 1, we present our estimates of the control means and average treatment effects of the chocolate scent, as computed with ordinary least squares. The direction of the treatment effect on total sales was positive but not statistically significant. None of our estimates of treatment effects are statistically significant at the two-tailed *p* < .10 level. Our findings are not substantively altered by adjusting for time trends.

**Table 1:** Estimated effects of chocolate scent on bookstore sales

Outcome	Unadjusted estimates	Adjusted for time trends
*Total sales*		
Control mean	575.4 (SE = 34.7)	593.5 (SE = 30.4)
Treatment effect	36.6 (SE = 51.6)	2.7 (SE = 41.7)
*Bulk sales*		
Control mean	72.0 (SE = 13.4)	71.2 (SE = 16.4)
Treatment effect	21.8 (SE = 17.5)	23.2 (SE = 21.2)
*Café sales*		
Control mean	334.7 (SE = 15.2)	336.5 (SE = 12.6)
Treatment effect	−5.5 (SE = 21.1)	−8.9 (SE = 16.2)
*Book sales*		
Control mean	168.7 (SE = 22.5)	185.7 (SE = 22.9)
Treatment effect	20.4 (SE = 32.0)	−11.6 (SE = 32.5)

Estimates of control means and average treatment effects of chocolate scent on total sales, bulk coffee bean/loose tea/spice sales, café sales, and book sales in Canadian dollars. All estimates computed using ordinary least squares regression with robust standard errors. Unadjusted estimates computed without covariates. Adjusted estimates computed using a linear control for the day of experiment and fixed effects for the day of week, all mean-centered

It is possible that when the consumer’s choice set includes food items such as pastries and coffee beans, these food items should be considered a product-type thematically congruent with a chocolate scent, and books a product-type thematically incongruent with a chocolate scent. In this case, countervailing effects of the sort Schifferstein and Blok test for and Doucé et al. suggest as an implication of their findings may be present, increasing the likelihood of purchasing a scent-congruent product type (food items) over a scent-incongruent product type (books). More precisely, a café on premise offers consumers an opportunity to purchase a product in the same *domain* as the appetizing scent (Li 2008).^2^ The presence of within-domain products from a café area (food items) could result in devaluation of the primary but out-of-domain retail products offered by the bookstore (books) (Brendl et al. 2003).

To test for such countervailing effects, we examine café sales and bulk sales relative to book sales. In all three outcome sub-categories—café sales, bulk sales, and book sales—the ambient chocolate scent treatment produced null results, providing neither evidence that a chocolate scent increased sales of a within-domain product (food items), nor evidence that the chocolate scent induced a preference for within-domain products over out-of-domain products (books). The direction of the treatment effect on café sales was slightly negative, though not statistically significant (average estimated decrease of 8.9CAD from a control average of 336.5CAD, two-tailed *p* = .59) and the direction of treatment on bulk coffee bean/loose tea/spice sales was slightly positive, though not statistically significant (average estimated increase of 23.2CAD from a control average of 71.2CAD, two-tailed *p* = .29).

## Conclusions

The aim of this study was to assess the generalizability of an effect of chocolate scent on product sales in a bookstore. With a slight alteration to the experimental setting—a bookstore with an adjoining café area—we find no effect of an ambient chocolate scent on sales. We find no effect within subset categories of sales, nor evidence of a countervailing effect on purchases of within-domain and out-of-domain products.

The lack of a detectable effect in our study could result from a number of different root causes that are inseparable without further testing. Our finding could indicate that the association between chocolate scent and increased book sales is spurious. On the other hand, these null results could indicate that book sales in the baseline condition have already been boosted by scents from the café area, rendering the chocolate scent treatment ineffectual in a setting where pleasant food-related scents naturally occur. In addition to the primary variation of interest in this extension—the presence of a café area adjoining the bookstore—a number of secondary features that differed between the two studies could account for the lack of a detectable effect in our study. For example, perhaps the effect of ambient scent on book sales is limited to larger, chain bookstores and does not affect sales in small, independent bookstores; or perhaps the effect is brought on only by atomized diffusion of a liquid scent and is diminished by the presence of real melted chocolate. Finally, it is possible that the effect of chocolate scents was small, and that our study is not well powered enough to detect it. Further interventions, in varied settings, could contribute to a meta-analytic understanding of the effects of chocolate scents on product sales.

Our study illustrates the value of examining the generalizability of experimental findings. These results do not preclude the existence of an effect of chocolate scent on purchasing behavior, but rather suggest the need for additional study in varied contexts.

## Footnotes

1 Considering atmospheric effects studies generally, Herrington (1996) also conducts a replication, testing the effects of supermarket music volume (replicating Smith and Curnow 1966) and tempo (replicating Milliman 1982).

2 Following Li’s definition of “domain”, *within*-*domain* products are those that can satisfy the specific physiological need targeted by the appetitive stimulus.
